# Free Floating Thrombus in Carotid Artery in a Patient with Recurrent Strokes

**DOI:** 10.1155/2017/4932567

**Published:** 2017-01-10

**Authors:** Moni Roy, Ashish Kumar Roy, Jeffrey R. DeSanto, Murad Abdelsalam

**Affiliations:** ^1^Department of Internal Medicine, University of Illinois College of Medicine, OSF Saint Francis Medical Center, 530 NE Glen Oak Avenue, Peoria, IL, USA; ^2^Department of Cardiology, St. Joseph Mercy Oakland Hospital, 44405 Woodward Avenue, Pontiac, MI, USA

## Abstract

We present a case of 72-year-old male with reported past medical history of recurrent transient ischemic attacks (TIAs) presenting with myriad of neurological symptoms. Patient was transferred from outlying hospital with complaints of right sided facial droop and dysarthria. Computed tomography angiography (CTA) showed high grade proximal left internal carotid artery (ICA) stenosis along with interesting finding of a free floating thrombus (FFT) in the left ICA. After discussion with the neurosurgical team, our case was treated conservatively with combination of antiplatelet therapy with Aspirin and anticoagulation with Warfarin without recurrence of TIAs or strokes on six-month follow-up.

## 1. Introduction

Free floating thrombus (FFT) in the carotid artery is an uncommon finding and rarely reported in the literature [1]. The treatment options for these patients include medical management or surgical interventions, both of which have been reported to have similar outcomes [2].

## 2. Case Presentation

**Figure 1:**
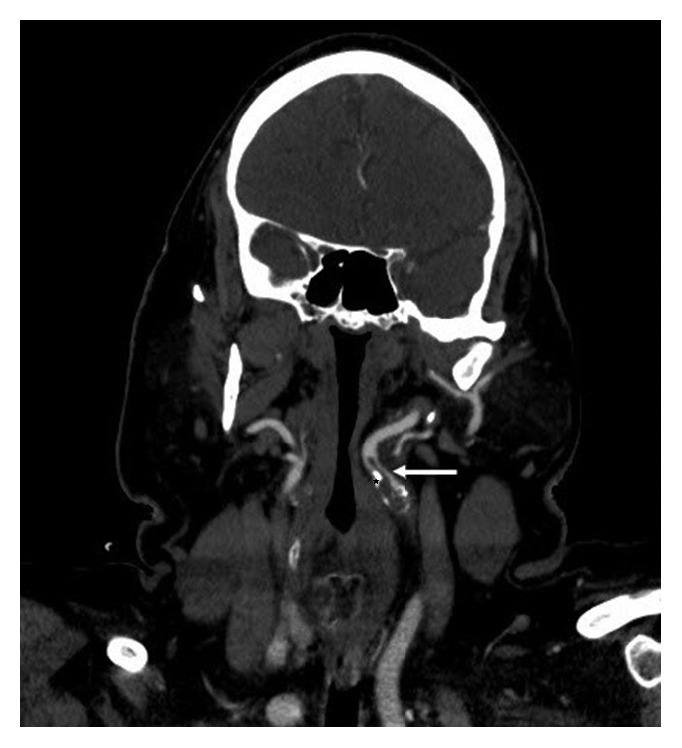
Coronal reformatted view from CT angiogram shows an intraluminal filling defect within the proximal left internal carotid artery consistent with thrombus (white arrow). There is also adjacent calcified atheromatous plaque (black asterisk). The internal carotid arteries are tortuous and deviate medially into the retropharyngeal space.

**Figure 2:**
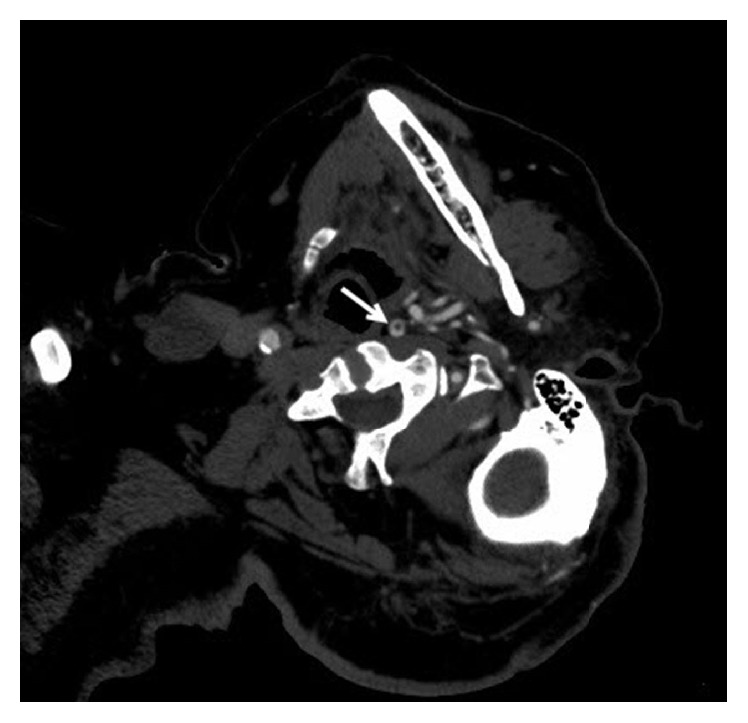
Axial image from CT angiogram demonstrates free floating thrombus within the internal carotid artery lumen “donut sign” (white arrow).

**Figure 3:**
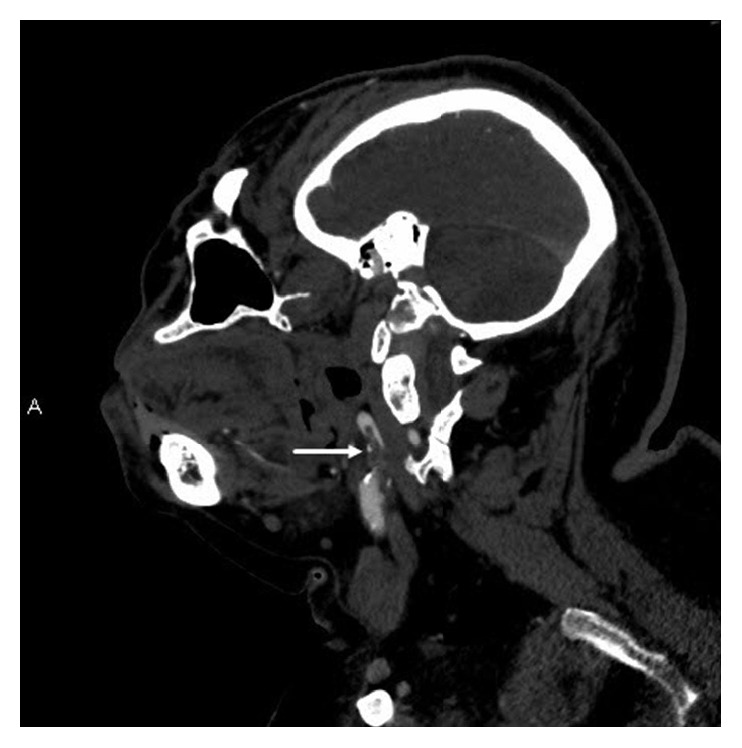
Sagittal oblique reformatted view from CT angiogram again depicts the intraluminal thrombus within the left internal carotid artery (white arrow).

A 72-year-old Caucasian male with past medical history of coronary artery disease, diabetes mellitus, hypertension, and hyperlipidemia was transferred to our facility with acute right sided facial droop and dysarthria. Initially, patient experienced gradual onset right lower extremity weakness followed by slurring of speech. Over the next day his symptoms progressed to right sided hemiplegia, dysarthria, and behavioral disturbances with emotional liability. Family reported history of cerebrovascular accidents in the past with no residual symptoms. Magnetic resonance imaging (MRI) done at outlying hospital showed old infarcts in right cerebellum, left parietal lobe, left thalamus, bilateral occipital lobes, and bilateral basal ganglia. Further workup at our facility included echocardiogram, which was normal. Cardiac telemetry monitoring revealed no arrhythmia. Prior carotid duplex from four years ago showed less than 50% stenosis of both carotid arteries and no repeat carotid duplex was done on admission. During his hospital stay, the right sided hemiplegia showed significant improvement on the next day of admission. However, on day three of hospitalization, he had an acute worsening of his right sided weakness. Stroke alert was called and emergent computed tomography angiography (CTA) of head and neck was ordered. CTA imaging showed 90% stenosis of proximal left internal carotid artery (ICA) with free floating thrombus (FFT) within the lumen along with adjacent calcified atheromatous plaque. Left paramedian pons hypodensity consistent with acute ischemia was a new finding. Occlusion of right vertebral artery at origin with collaterals from occipital artery and occluded posterior inferior cerebellar artery (PICA) were noted (Figures 1–3).

## 3. Treatment

Patient was transferred to neurology intensive care unit and was started on heparin drip. Neurosurgery and vascular surgery services were consulted. After discussions with patient and family; medical management was decided with both anticoagulation and antiplatelet therapy. He was started on Aspirin and Warfarin with international normalized ration (INR) target of 2-3.

## 4. Outcome and Follow-Up

Patient was transferred to inpatient stroke rehabilitation unit for three weeks of occupational and physical therapy. He later was safely discharged home on Aspirin and Warfarin for long term anticoagulation. At six months of follow-up, patient did not have recurrence of any neurological deficits and showed good residual motor function.

## 5. Discussion

Free floating thrombus is an uncommon entity with variable reported incidence depending on method of imaging used. Incidence has been reported to be as low as 0.05% in a retrospective study with ultrasonography used for carotid artery imaging [1]. On the other hand, Buchan et al. detected FFT in 1.45% (29/2,000) angiograms of patients with high- or moderate-grade ICA stenosis [3].

There are multiple and diverse causes of carotid FFT formation. Atherosclerosis has been reported to be the most common etiology. Carotid stenosis in itself with altered blood flow around the area of stenosis would cause increased risk of FFT. As per our literature review, no direct correlation between the degree of preexisting stenosis and risk of FFT has been reported. Our patient had a carotid ultrasound four years ago with less than 50% stenosis in bilateral carotid arteries, classified as mild stenosis per North American Symptomatic Carotid Endarterectomy Trial (NASCET) [4]. Our patient had a progression of stenosis from less than 50% to 90% over 4-year period. Smaller studies have been done to evaluate the progression of carotid stenosis over years, though no clear recommendations on surveillance exist. Johnson et al., in study of 232 patients with less than 80% stenosis, reported that 23% over 10 years progressed to severe stenosis (defined as 80–99%) [5]. Park et al. studied the natural history of asymptomatic moderate carotid artery stenosis and reported 32% of cases progressed to severe stenosis but only 97% became symptomatic [6].

FFT usually presents with acute neurological deficit. Bhatti et al. reported 92% of cases with neurological symptoms and 4% were asymptomatic [2]. Similar results were reported by Ferrero et al. where 14 out of 16 cases over a period of 9 years were symptomatic [7]. Our patient was symptomatic at time of presentation with neurological deficits. Also his reported history of TIAs prior to admission was likely clinical manifestation of developing FFT.

Different imaging modalities have been used to diagnose FFT. Ferrero et al. in a single center study reported that duplex scan and digital subtraction angiography (DSA) had sensitivity of 62.5% and 100%, respectively [7]. The current American Heart Association for acute neurovascular imaging recommends CTA as the preferred modality for imaging the vasculature in acute stroke or TIA. There are well defined signs on CTA such as the “donut sign” described by Menon et al. [8] (Figure 2). Jaberi et al. in their prospective single center study showed a cranial-caudal measurement threshold of more than 3.8 mm on a CTA was highly specific and sensitive for FFT diagnosis [9]. Though smaller studies have supported the use of DSA, CTA appears to be the most common imaging used due to easy availability. CTA has also been further studied to report specific radiological signs to diagnose this condition. Larger studies to determine the gold standard imaging modality in FFT need to be done. In contrast, our case underwent emergent CT head with CTA that did show a FFT in left ICA (Figures 1–3).

In the published data, different types of treatments have been described with good results: medical management, medical management with deferred surgery, urgent endarterectomy, or endovascular treatment [2, 3, 8]. Bhatti et al. studied 145 patients with FFT, and follow-up data on radiological evaluation after treatment was available on 28 out of 33 patients treated with anticoagulation with or without antiplatelet therapy. Complete dissolution of FFT on repeat imaging after completion of therapy was reported in 86% when medically treated. The duration of therapy ranged from 2 weeks to 24 weeks. In their study, 94 patients underwent surgical treatment with carotid stenting, carotid bypass, or CEA. In this study, results of patients' outcome appeared similar for medical and surgical intervention. Gülcü et al. did another study that supported medical management, with 34 out of 37 medically treated patients having resolution of thrombus, and also showed that emergent surgical intervention is not always needed [10]. Newer endovascular techniques such as self-expanding stent placement and suction thrombectomy to prevent embolic complications from FFT were reported by Park et al. and Parodi et al. [11, 12]. However, at this time no randomized trial exists to support medical versus surgical treatment. Our patient was treated using medical approach with heparin drip initially and later continuation of anticoagulation with Coumadin with antiplatelet therapy. Our patient had favorable outcome with no recurrence of neurological deficit over 6 months of follow-up.

## 6. Learning Points/Take Home Messages

1. A free floating thrombus is an elongated thrombus attached to the arterial wall with circumferential blood flow at the distal aspect.
2. Atherosclerosis is reported to be the most common etiology. The most likely pathogenesis of a free floating thrombus is ruptured atherosclerotic plaque that increases the risk for thrombus formation at the site.
3. Most patients with FFT present with acute and fluctuating neurological symptoms. Artery-to-artery embolism leads to acute neurological deficits.
4. No direct correlation between the degree of preexisting carotid artery stenosis and risk of FFT has been reported.
5. No clear recommendations exist for medical management (antiplatelet and anticoagulation) versus surgical management (including carotid artery stenting, bypass, or CEA), both of which have shown similar outcomes.

## References

[1] Arning C., Herrmann H. D. (1988). Floating thrombus in the internal carotid artery disclosed by B-mode ultrasonography. *Journal of Neurology*.

[2] Bhatti A. F., Leon L. R., Labropoulos N. (2007). Free-floating thrombus of the carotid artery: literature review and case reports. *Journal of Vascular Surgery*.

[3] Buchan A., Gates P., Pelz D., Barnett H. J. M. (1988). Intraluminal thrombus in the cerebral circulation implications for surgical management. *Stroke*.

[4] Eliasziw M., Rankin R. N., Fox A. J., Haynes R. B., Barnett H. J. M. (1995). Accuracy and prognostic consequences of ultrasonography in identifying severe carotid artery stenosis: North American Symptomatic Carotid Endarterectomy Trial (NASCET) Group. *Stroke*.

[5] Johnson B. F., Verlato F., Bergelin R. O., Primozich J. F., Strandness D. E. (1995). Clinical outcome in patients with mild and moderate carotid artery stenosis. *Journal of Vascular Surgery*.

[6] Park Y.-J., Kim D.-I., Kim G.-M., Kim D.-K., Kim Y.-W. (2016). Natural history of asymptomatic moderate carotid artery stenosis in the era of medical therapy. *World Neurosurgery*.

[7] Ferrero E., Ferri M., Viazzo A. (2011). Free-floating thrombus in the internal carotid artery: diagnosis and treatment of 16 cases in a single center. *Annals of Vascular Surgery*.

[8] Menon B. K., Singh J., Al-Khataami A., Demchuk A. M., Goyal M. (2010). The donut sign on CT angiography: an indicator of reversible intraluminal carotid thrombus?. *Neuroradiology*.

[9] Jaberi A., Lum C., Stefanski P. (2014). Computed tomography angiography intraluminal filling defect is predictive of internal carotid artery free-floating thrombus. *Neuroradiology*.

[10] Gülcü A., Gezer N. S., Men S., Öz D., Yaka E., Öztürk V. (2014). Management of free-floating thrombus within the arcus aorta and supra-aortic arteries. *Clinical Neurology and Neurosurgery*.

[11] Park J. W., Lee D. H., Choi C. G., Kim S. J., Suh D. C. (2012). Various endovascular approaches to the management of free floating carotid thrombi: a technical report. *Journal of NeuroInterventional Surgery*.

[12] Parodi J. C., Rubin B. G., Azizzadeh A., Bartoli M., Sicard G. A. (2005). Endovascular treatment of an internal carotid artery thrombus using reversal of flow: a case report. *Journal of Vascular Surgery*.

